# Enhanced Neurokinin-1 Receptor Expression Is Associated with Human Dental Pulp Inflammation and Pain Severity

**DOI:** 10.1155/2021/5593520

**Published:** 2021-05-05

**Authors:** Riffat Mehboob, Sana Hassan, Syed Amir Gilani, Amber Hassan, Imrana Tanvir, Humaira Waseem, Asif Hanif

**Affiliations:** ^1^Research Unit, Faculty of Allied Health Sciences, The University of Lahore, Lahore, Pakistan; ^2^University Institute of Public Health, Faculty of Allied Health Sciences, The University of Lahore, Lahore, Pakistan; ^3^Department of Pathology, King Abdul Aziz Medical University, Rabigh, Saudi Arabia

## Abstract

Substance P (SP) is a peptide involved in many biological processes, including nociception and inflammation. SP has a high affinity for its receptor neurokinin-1 (NK-1R). SP/NK-1R complex plays a major role in the interactions going on during the onset of dental pain and inflammation. *Objective*. To identify the expression of NK-1R in healthy and inflamed human dental pulp, as well as to identify any association with severity of dental pain. *Methods*. This case-control study included ten irreversibly inflamed samples of dental pulp, which were extirpated from patients presenting with chief complaint of dental pain due to caries. Ten healthy pulps, extirpated from those teeth which were indicated for extraction due to orthodontic reasons, were used as the control group. Visual analog scale (VAS) and modified McGill Pain Questionnaire were used to assess the characteristic and severity of pain. Immunohistochemical study was performed using monoclonal antibodies against NK-1R. *Results*. The results showed that the NK-1R was expressed intensely in patients with higher pain score. The mean pain score in cases was 7.0 ± 2.0. The healthy dental pulps had negative or mild NK-1R staining of +1 intensity. The NK-1R score in cases was 2.4 ± 0.516 and 0.2 ± 0.4216 in controls. There was significant difference in NK-1R score between both groups (*p* value <0.05). There was a strong positive correlation between the pain score and NK-1R expression score. As the pain increased, the NK-1R expression score was also increased (0.95^∗∗^, *p* value 0.000). *Conclusions*. NK-1R is overexpressed in inflamed dental pulp. SP/NK-1R modulation may provide a novel approach for the treatment of pulpal inflammation and pain.

## 1. Introduction

Dental pain perception is due to an inflammatory reaction going on inside the tooth pulp [1]. Neuropeptides are now known to be major indicators of the inflammatory process in peripheral tissues. Neuropeptides take part in the process of transmission and modulation of pain and inflammatory process. These neuropeptides include neurokinin A (NK-A), calcitonin gene-related peptide (CGRP), neuropeptide Y (NP-Y), vasoactive intestinal polypeptide (VIP), and substance P (SP), among others [2].

Prior studies have demonstrated SP to be involved in both inflammation and pain [3]. SP is abundantly present in the fibers that innervate the dentin and dental pulp. As the inflammatory processes begin, the amount of SP released by each sensory fiber is further increased, which perpetuates the vicious circle that is underlying inflammation [4]. SP binds to its receptor NK-1R preferentially and exerts all its biological actions by binding to this high-affinity G-protein-coupled receptor. These receptors are located on many inflammatory cells, such as macrophages and mast cells, and also on other connective-tissue cells [5]. SP can also interact with NK-2 and NK-3 receptors, if present in higher concentrations. There are reports suggesting the presence of NK-1R, as G-protein-coupled receptor subunits have been identified in normal human dental pulp [6, 7].

This molecule's production and release is significantly enhanced when the dental pulp is stimulated by multiple factors such as noxious, thermal, mechanical, and chemical triggers [8]. The increase in blood vessel permeability, thus enabling plasma extravasation and degranulation of mastocytes, is due to induced vasodilatation caused by the interaction between SP and its receptors. Histamine is released from mastocyte granules, which in turn activates and enhances vascular mechanisms.

Lymphocytes, granulocytes, and macrophages have SP receptors, and cytokine production is induced by stimulation of these cells [9]. The interaction of SP with mast cells describes the reason for the enhanced vascular permeability and blood pressure due to induction of release of histamine. Moreover, the macrophages, lymphocytes, and granulocytes contain sites of NK-1R, and these cells are stimulated by SP and induce the production of proinflammatory mediators and cytokines. In addition, SP serves as a powerful chemical agent, which results in further incorporation of inflammatory cells in the pulp tissue.

There is an increased sensitivity towards pain because of dramatic sensitizing of nociceptors due to incorporation of more inflammatory and nociceptive mediators, also resulting in stimulation and abundant release of SP not only in the spinal cord but also within the pulpal tissue [10, 11]. SP affects the dental pulp in many ways. Multiple studies have marked the presence of NK receptors in rodent and human teeth. Animal studies established the pattern of expression of the tachykinin receptors NK-1, NK-2, and NK-3 in different types of epithelial cells, fibroblasts, hard tissue cells, endothelium, and blood vessel walls in teeth and surrounding oral tissues [12].

It has been documented that NK-1R and NK-2R are present both on ameloblasts (enamel-forming cells) and odontoblasts. As predicted, there is an abundance of NK-1Rs on smaller blood vessels, and capillaries and both NK-1R and NK-2R are densely distributed on the capillary plexus underneath dentin [13]. Although, presence of NK-Rs has been reported in human pulp tissue, but the methods used for their evaluation like radio-receptor assay were not precise enough to measure which type of receptor (NK-1, NK-2, or NK-3) was primarily present [9]. However, there is no exact evidence of the sequence and expression of NK-1R in healthy and inflamed human dental pulp while transitioning during different stages of inflammation. This study was thus aimed at comparing expression of particularly NK-1R in human pulp tissue extirpated from teeth clinically diagnosed with irreversible pulpitis to those of healthy teeth in association with severity of pain.

## 2. Methodology

### 2.1. Tissue Collection

The study was approved by Ethical Review Committee of The University of Lahore and performed in accordance with the guidelines of the Declaration of Helsinki for Human Research [14]. Complete history was taken from all the patients after taking written informed consent. Twenty pulp samples were obtained from 2 groups of patients of both gender with age ranging between 15 and 35 years [15]. After fulfilling the clinical diagnostic criteria [16], group 1 (cases) included inflamed samples of dental pulp obtained from teeth clinically diagnosed with irreversible pulpitis, and these patients presented in the outdoor patient department (OPD) with chief complaint of dental pain. Group 2 (controls) included pulp samples obtained from healthy teeth with fully developed roots, which needed extraction due to orthodontic reasons, and these patients had no complaint of pain [11]. Both of these groups had no history of antibiotic administration, and they were systemically healthy. Smokers, pregnant, and patients with history of trauma to orofacial region or a TMJ surgery were excluded from this study. Any patient who was suffering from irreversible pulpitis but did not complain of dental pain was excluded from group 1, and patient complaining of dental pain associated with the tooth to be extracted was also excluded from group 2. Group 1 was given the VAS and modified McGill Pain Questionnaire to assess the characteristics and intensity of dental pain [17].

Teeth belonging to group 1 were anesthetized by 1.8 ml 2% lidocaine, injection technique was infiltration for maxillary teeth, and inferior alveolar nerve block was given for mandibular teeth. To obtain pulp sample from group 1, access opening was made and pulp extirpation was done using a no. 15 barbed broach [4]. Immediately after the extirpation, the tissue was placed in 10% neutral buffered formalin, embedded in paraffin, and cut with a microtome to a thickness of 3-4 *μ*m. For each tissue sample, three different depths of cut were made, which were first stained with Hematoxylin-Eosin (H&E). Observation of H&E stained sections involved determination of the form and intensity of inflammation.

For the group of healthy pulps, the teeth were anaesthetized and extracted in the same manner. 5.25% sodium hypochlorite was used to wash the teeth, soon after extraction in order to eliminate remnants of periodontal ligament which can result in contamination of the pulp sample. The sectioning of teeth was done by using a cylindrical diamond bur in a high-speed hand piece with simultaneous irrigation with saline solution. Extirpation of pulp was done by a barbed broach.

### 2.2. Immunohistochemical (IHC) Staining Protocol

3-4 *μ*m thick sections of the dental pulp were mounted on 3-aminopropyltriethoxysilane- (APES-) coated slides, deparaffinized in xylene, and rehydrated via graded ethanol solutions. Then, sections were rinsed with distilled water and washed three times with PBS (pH 7.4). Heat-induced pretreatment for antigen retrieval (sections were immersed in a 10 mmol/l citrated buffer, pH 6.0, at 60°C for 5 min) was carried out prior to incubation with the primary antibody. The endogenous peroxidase activity was inhibited by incubation of the samples with 0.3% hydrogen peroxidase in methanol for 30 minutes at the temperature of 4°C. After blocking the nonspecific reactions with 10% normal rabbit serum, the sections were incubated with the primary antibody against NK-1R for two hours.

### 2.3. Evaluation of Immunohistochemical Staining

The slides were examined by light microscopy using an Olympus BX40 microscope (Artisan Scientific, Champaign, Illinois, USA) at magnification of 400x. The product of the immunohistochemical reaction was detected in the cytoplasm of the endothelial cells, fibroblasts, and inflammatory cells. NK-1R staining was identified by displaying brown color, ranging from light brown to dark brown. The cytoplasmic staining intensity was scored semiquantitatively, according to a previously described method [18], as follows:

0–10% = negative

10–30% = 1 + (weak staining)

30–60% = 2 + (moderate staining)

60–100% = 3 + (strong staining)

## 3. Statistical Analysis

Data were analyzed by using SPSS 25.0. All the quantitative variables were presented by frequencies and quantitative variables in mean ± SD. Independent sample *t*-test was applied to check the mean difference of pain score and NK-1 between cases and controls. Pearson correlation was applied to check the relationship between pain and NK-1R scoring.

## 4. Results

Levels of NK-1R were much higher in the irreversibly inflamed pulp versus the healthy pulp. Patients with a diagnosis of healthy pulp had a pain rating on the visual analog scale (VAS) of 0. Patients with a diagnosis of irreversible pulpitis had a lowest pain rating score of 4 out of a maximum score of 10. Scores above 5 are associated with moderate to very severe pain. This study included 10 cases of irreversible pulpitis (7 were acutely inflamed cases, and 3 were chronic cases as diagnosed clinically). A positive correlation between expression of NK-1R, level of inflammation, and pain score was observed in 7 cases clinically diagnosed as acute pulpitis. These cases showed higher pain score ranging from 6 to10 and mild to severe levels of inflammation histologically. The NK-1R expression also ranged from moderate to intense staining. Level of inflammation and staining increased with the increasing pain score. On the other hand, 3 cases clinically diagnosed as chronic pulpitis showed moderate levels of inflammation and NK-1R expression. However, the pain score for chronically inflamed cases was moderate (6 as rated on VAS) as compared to acutely inflamed cases where pain score ranged from moderate to severe (above 6 on VAS).

Table 1 shows the demographic variables. The mean age of patients was 21 ± 6.665 years. There were 5 males and 5 females in group 1 (cases) and 2 males and 8 females in group 2 (controls), and majority were single (cases = 5 and control = 10), qualification level of intermediate (cases = 5 and control = 2), using no medication for pain (cases = 2 and control = 10), good oral hygiene (cases = 7 and control = 10), negative NK-1R staining (cases = 0 and control = 8), and (cases = 4 and control = 0) had severe staining.

According to Table 2, the mean pain score in cases was 7.0 ± 2.0. The NK-1R score in cases was 2.4 ± 0.516 and 0.2 ± 0.4216 in controls. There was significant difference in NK-1R score between both groups (*p* value <0.05). There was a strong positive correlation between the pain score and NK-1R. As pain increased, the NK-1R score also increased (0.95^∗∗^, *p* value 0.000) (Table 3).

**Figure 1:**
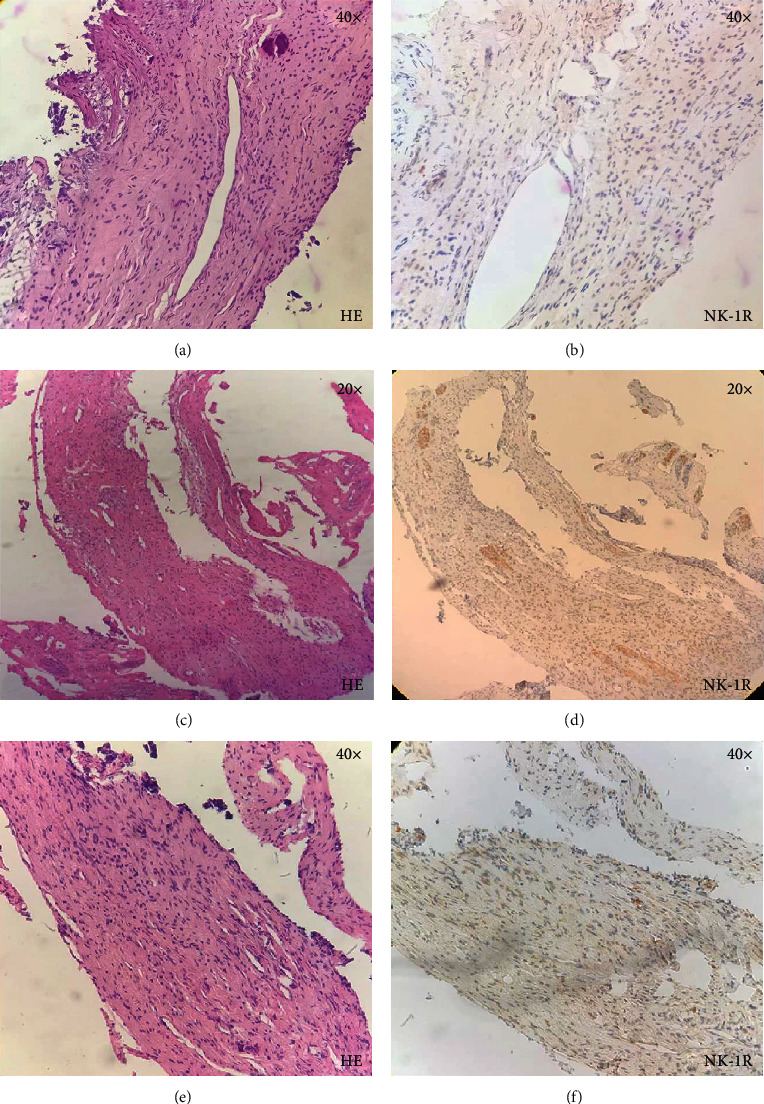
Histological sections of healthy dental pulp.

**Figure 2:**
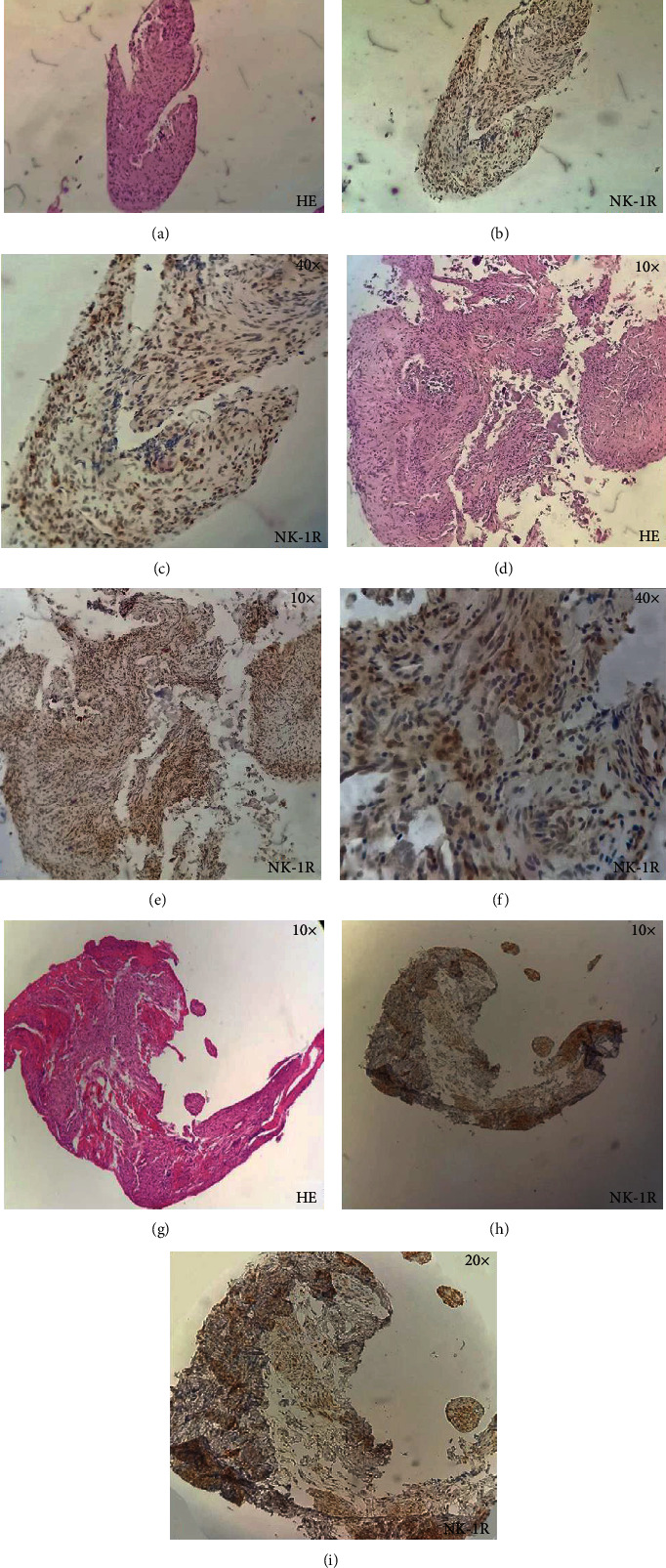
Histological sections of inflamed dental pulp.

Figure 1 is representing the preservation of normal morphology of dental pulp. Figures 1(a), 1(c), and 1(e) show H/E staining, consisting primarily of mesenchymal cells, fibroblasts, and some macrophages, exhibiting a stellate morphology. Figures 1(b), 1(d), and 1(f) show NK-1R staining. Figures 1(b) and 1(d) show negative staining, and Figure 1(f) shows mild staining. Figures 2(a), 2(d), and 2(g) show vascular congestion, and there is presence of diffuse inflammatory infiltrate, predominantly formed by lymphocytes and plasma cells. Figures 2(b), 2(e), and 2(h) are showing intense NK-1R staining in inflamed dental pulp at 20x and Figures 2(c), 2(f), and 2(i) at 40x.

## 5. Discussion

Induction of inflammatory reaction is due to increased amount of SP in peripheral tissues including the dental pulp, but the regulatory effects can only be witnessed in target tissues if there is enhanced receptor signalling [19]. Therefore, the objective of this study was to compare SP receptor, NK-1R expression in pulpal tissue with clinical diagnosis of irreversible pulpitis, to that of in healthy pulp. The mechanism by which pain arises when an inflammatory reaction is going on within the dental pulp is not completely understood.

Studies reveal that irreversible pulpitis is associated with different expression of various biomarkers, as compared to noninflamed controls. One study reported findings in rat's dental pulp that immunoreactions for NK-1R were detectable in nerve terminals associated with cytoplasmic processes of the odontoblasts [20]. Several other studies suggest that SP/NK-1R has a definitive role in inflammatory processes. This was confirmed due to considerably higher SP/NK-1R expression in the inflamed pulp [21, 22]. One study reported an 8-fold increase in levels of SP, and it was eminent in pulpal tissue clinically diagnosed with irreversible pulpitis versus that of clinically healthy pulp tissue [22]. Consequently, irreversible pulpitis is associated with momentous instigation of this peptidergic system but there is lack of sufficient data related to expression of NK-1R in human dental pulp during inflammation. Another study highlighted that SP, IL-8, and MMP-8 levels were found to be higher in pulpal samples from teeth with irreversible pulpitis, with higher pain scores than those with low pain scores [23].

Another study reported that NK-1R was elevated along with SP in gingival crevicular fluid around painful teeth [23]. Recently, the involvement of SP-NK-1R in oral pain and inflammation has also been reported in a study from Spain [24]. SP has been a major player in the neurogenic inflammation in the afferent neurons and may contribute to the pulpal disease. SP mRNA and protein were expressed by pulpal fibroblasts. N-1R mRNA was also detected in these fibroblasts. SP levels were also observed to be high as compared to the healthy pulps [25]. Only a single study has been performed previously, reporting the expression of SP receptor, NK-1R in human dental pulp. This study proved that SP receptor expression was present in all human pulp tissue samples, and there was an intense expression of NK-1R in the group of pulps from teeth clinically diagnosed with acute irreversible pulpitis [4]. Cytokine interleukin 1 (IL-1) has been observed to be main player in the periodontitis [26].

In a study conducted on 25 human dental pulps, a histological and radioimmunological assay was performed to correlate the concentration of prostaglandin E2 (PGE2) and grade of inflammation. The study confirmed an association, and PGE2 was reported as a marker to distinguish the reversible and irreversible pulpitis [27]. Results from our study correlate with prior statement, as there was an increase in expression of SP receptors during inflammatory processes, and this indicates SP active contribution in the development of pulpitis. Our study is first to demonstrate the comparative expression of NK-1R in healthy and inflamed human dental pulp in association with severity of dental pain. While these results cannot be inferred to address existing endodontic clinical problems, these results have clinical implications, as they have the potential for inspiring future research modalities when proposing alternative methods of biologic pulp therapy. Identifying SP receptor in human pulp, while focusing on its regulatory effects on immune reactions, specially taking into account its role in the induction of fibroblastic proliferation, is of utmost importance in order to broaden the scope of knowledge of biological action of these receptors.

## 6. Conclusions

The expression of NK-1R in human pulp tissue is remarkable during the process of pulpal damage caused by an inflammatory reaction. Considering the correlation between the symptomatic teeth with increased SP levels and patient VAS scores, SP/NK-1R modulation may provide a novel approach for the treatment of pulpal inflammation and pain. Definitely, familiarity with different processes involved in pulp inflammation is required in order to diagnose, plan, and formulate treatment modalities leading towards better management of the diseased pulpal tissue, thus not only maintaining its vitality but also preventing subsequent loss of resistance and retention of tooth.

## Figures and Tables

**Table 1:** Demographic variables.

Variable	Cases	Control	Total
Gender			
Male	5	2	7
Female	5	8	13
Marital status			
Single	5	10	15
Married	5	0	5
Qualification			
Illiterate	1	2	3
Middle	0	5	5
Intermediate	5	2	7
Bachelor	4	1	5
Use of medication to reduce pain			
No	2	10	12
Pain killer	8	0	8
Oral hygiene			
Poor	2	0	2
Good	7	10	17
Excellent	1	0	1
Diagnosis			
Acute pulpitis	7	0	7
Chronic pulpitis	3	0	3
NK-1 stain			
Negative	0	8	8
Mild	0	2	2
Moderate	6	0	6
Severe	4	0	4

**Table 2:** Mean difference of pain score and NK-1R between cases and controls.

Independent sample *t*-test						
	Group	*N*	Mean	Std. deviation	*t*-test	*p* value
Pain score	Case	10	7.00	2.000	11.068	0.000
	Control	10	.00	.000		
NK-1R scoring	Case	10	2.4000	.51640	10.436	0.000
	Control	10	.2000	.42164		

**Table 3:** Pearson correlation.

Correlations			
		Pain score	NK-1R scoring
Pain score	Pearson correlation	1	.955^∗∗^
	*p* value		.000
	*N*	20	20

^∗∗^Correlation is significant at the 0.01 level (2-tailed).

## Data Availability

The datasets used to support the finding of this study are available from the authors upon reasonable request.
